# Knowledge and acceptance of HPV vaccination and its associated factors among parents of daughters in Addis Ababa, Ethiopia: a community-based cross-sectional study

**DOI:** 10.1186/s13027-021-00399-8

**Published:** 2021-09-03

**Authors:** Nebiyu Dereje, Abigia Ashenafi, Anteneh Abera, Efrata Melaku, Kaleb Yirgashewa, Meron Yitna, Sarona Shewaye, Tsedenia Fasil, Yadel Yoseph

**Affiliations:** 1 Department of Public Health, Myungsung Medical College, P.O Box 14972, Addis Ababa, Ethiopia; 2 Department of Medicine, Myungsung Medical College, Addis Ababa, Ethiopia

**Keywords:** HPV, Knowledge, Vaccine acceptance, Cervical cancer, Ethiopia

## Abstract

**Background:**

Cervical cancer is one of the most diagnosed and deadly cancers in women globally. Though vaccination is an effective way to reduce cervical cancer, people’s knowledge and acceptance of the vaccination remains a challenge in low and middle-income countries. Therefore, the aim of this study was to assess the level of knowledge and acceptance of Human Papilloma Virus (HPV) vaccination and its associated factors among parents of daughters in Addis Ababa, Ethiopia.

**Methods:**

A community-based cross-sectional study was conducted among parents or guardians whose daughters are in the age group of 9–17 years and residing in Akaki-Kalty sub-city in Addis Ababa. A multistage sampling technique was used to select the study participants (n = 430). Face-to-face interview was conducted by using a structured questionnaire. Factors associated with the acceptance of HPV vaccination was identified by multivariable binary logistic regression and expressed by adjusted odds ratio (aOR), and respective 95% confidence interval.

**Results:**

Complete response was obtained from 422 (98.1%) of the participants and their mean age was 39.0 years (SD ± 9.9). Out of the study participants, 41.7% and 72.0% had poor knowledge on cervical cancer and HPV, respectively. More than a quarter (27.0%) of the participants has never heard about HPV vaccine. One-third (36.5%) of the participants had negative attitude towards the HPV vaccine. Overall, 94.3% of the study participants were willing to vaccinate their daughters for HPV. Vaccine acceptability was associated with higher monthly income (aOR = 2.48, 95% CI 1.08–6.34), good knowledge on HPV (aOR = 2.32, 95% CI 1.56–4.87) and the vaccine (aOR = 2.24, 95% CI 1.12–8.60), and positive attitude towards the vaccine (aOR = 5.03, 95% CI 1.63—9.56).

**Conclusions:**

The overall HPV vaccine acceptance was high. However, two out of five and one-thirds of the parents had poor knowledge on cervical cancer and negative attitude towards the HPV vaccine, respectively. Higher monthly income, good knowledge on HPV and the vaccine, and positive attitude towards the vaccine were associated with acceptance of HPV vaccination. To ensure sustainable acceptance of HPV vaccination, it is crucial to increase the community awareness in a sustainable manner.

## Background

Cervical cancer is a malignant tumor arising from the cells of the uterine cervix. The oncogenic human papillomavirus (HPV) infection is the main causative agent of cervical cancer [1, 2]. HPV-16 and 18 are the most important high-risk types, accounting for about 70% of cervical cancers worldwide [2, 3]. In a population-based study conducted in south central Ethiopia, the most frequently identified high-risk genotypes were HPV-16, 35, 52, 31 and 45 [4].

Worldwide, cervical cancer is the fourth most commonly diagnosed malignancy and the second deadly cancer in women creating a major public health concern globally. In 2020, the global estimate was 604,000 new cases resulting in 342,000 mortalities [5]. In Ethiopia, 6294 new cases and 4884 deaths occur annually. As of 2020, the age specific incidence and mortality rate was 21.5 and 16 deaths per 100,000 females, respectively [2].

HPV vaccination plays a fundamental role in the prevention of cervical cancer. The first safe and efficacious HPV vaccine was developed in 2006. It was a quadrivalent Gardasil/Silgard, that targets HPV-6, 11, 16, and 18, and the bivalent Cervarix vaccine targeting HPV-16 and 18 [6, 7]. Reduced HPV prevalence of 73–85% and 41–57% decline in high grade cervical lesions has been reported from countries with high coverage of HPV vaccination [1, 7, 8]. Ethiopia launched HPV vaccine for the first time in 2018 for all 14 year old girls through a school-based approach and in health centers [9]. Nevertheless, studies reported that unsubstantiated rumors about side effects or adverse outcomes that are not related to the vaccine negatively impacted public trust and have led to suspension of the HPV vaccination program [10–16].

Since HPV vaccination is a new program in Ethiopia, there are only few studies conducted on the knowledge and acceptability of HPV vaccine. In a study conducted in Gondar (North-west Ethiopia), 81.3% accepted to vaccinate their daughters for HPV [10]. Another study in Bench-Sheko zone, south-west Ethiopia, reported that 79.5% of parents have accepted to vaccinate their daughters for HPV [15]. Both studies revealed that parental acceptance to vaccinate their daughters is affected by the knowledge and attitude of the parents. To our knowledge, however, there is no study on the topic from Addis Ababa, the capital city of Ethiopia, where the level of health literacy of the population is expected to be higher than the other parts of the country.

Knowledge on cervical cancer, HPV infection, HPV vaccine and national HPV vaccination program plays a major role in acceptance of the vaccine [17–19]. However, community perception of HPV vaccine is not well assessed in many low and middle-income countries including Ethiopia. For the effective scale-up of cervical cancer prevention programs, barriers to the acceptance of vaccination need to be assessed and addressed. Therefore, the aim of this study was to assess knowledge and the level of HPV vaccine acceptability and its associated factors among parents or guardians of daughters in Addis Ababa, capital city of Ethiopia.

## Methods

### Study setting and design

A community-based cross-sectional study was conducted among parents or guardians with daughters in the age group of 9–17 years in Akaki Kality sub-city, Addis Ababa, from January 20–31, 2021. Akaki Kality sub-city is one of the major sub-cities in Addis Ababa comprising of 13 districts. The estimated population was about 220,740 with the female population of 114,095.

### Study population

The study population was selected parents or guardians with daughters in the age group of 9–17 years who were permanent residents (lived at least six months in the sub-city prior to the survey) of Akaki Kality sub-city, Addis Ababa.

### Sample size and sampling procedures

Four hundred thirty (430) samples were estimated by using single population proportion formula by taking 95% confidence interval, 4% margin of error, 79.5% proportion of HPV vaccine acceptance in Bench-Sheko zone, south-west Ethiopia [15] and adding up 10% non-response rate. Two-stage sampling technique was employed to recruit the participants for the study. In the first stage, out of the 13 districts in the sub-city, three of them were selected randomly (lottery method). The total sample was allocated proportionally to the districts. Then, the households from each district were selected by employing a systematic random sampling (sampling interval = every 4th house). If there was more than one parent in a selected household, one of them was randomly selected by lottery as a study participant. Parents or guardians with daughters in the age group of 9–17 years who permanently resided in Akaki Kality sub-city (for at least 6 months) and have given written consent were included in the study. Those participants who were mentally incompetent or severely ill and who were not around during the data collection time after two separate visits were excluded from the study. Whenever there was no eligible individual to be included in the study, the next household was replaced instead.

### Data collection procedure and variables

Data were collected using a questionnaire which was adapted after a thorough literature review of previously conducted studies [10, 14, 20, 21]. The questionnaire was translated to Amharic to avoid language barrier. The questionnaire contains information on the socio-demographic characteristics of the participants, knowledge towards cervical cancer and HPV, attitude and acceptability of HPV vaccination. Face-to-face interview was administered to collect data from the study participants.

Guardian was defined as a person who has the legal authority (and the corresponding duty) to care for the personal and property interests of a minor daughter. The dependent variable of the study was acceptability of HPV vaccination which was assesses by using a question “Are you willing to vaccinate your daughter for HPV vaccination which can protect against HPV infection? [10, 22]. The independent variables assessed in the study includes monthly income, marital status, educational status of the parents, knowledge on cervical cancer, knowledge of HPV, knowledge of vaccine, and attitude towards the vaccine. Knowledge of cervical cancer was assessed by 11 yes or no knowledge-based questions. Then, the knowledge score was categorized in two as below or above the mean score. The mean and below knowledge score was considered as poor knowledge while above the mean was considered as good knowledge [14, 20, 23]. Knowledge of HPV was assessed by 4 yes or no knowledge-based questions. Then, the knowledge score was categorized in two as below or above the mean score. The mean and below knowledge score was considered as poor knowledge while above the mean was considered as good knowledge [14, 20, 23]. Knowledge of HPV vaccine was assessed by 8 yes or no knowledge-based questions. Then, the knowledge score was categorized in two as below or above the mean score. The mean and below knowledge score was considered as poor knowledge while above the mean was considered as good knowledge. Attitude for HPV vaccine was assessed by 10 questions which was in three Likert scale (agree, neutral, disagree) then mean score was calculated. Then, the attitude score was categorized in two as below or above the mean score. The mean and below attitude score was considered as negative attitude while above the mean was considered as positive attitude [14, 20, 23].

### Data management and analysis

Data was coded and entered into SPSS for windows version 25.0 for analysis. Frequency and proportions were used to summarize categorical variables, whereas mean and standard deviation were used to summarize continuous variables.

Multivariable binary logistic regression analysis was carried out to identify factors associated with HPV vaccine acceptance, as expressed by adjusted odds ratio (aOR) along with its respective 95% confidence interval (CI). Variables with *P* value < 0.25 in bivariate analysis were considered for multivariable analysis. Variables having *P* value < 0.05 were considered statistically significant. The goodness of model was assessed by the Hosmer Lemeshow test, and it was not significant (*P*-value = 0.83) and there was no multicollinearity (Variance inflation factor = 2.5 and the tolerance test = 0.4).

### Ethical considerations

Ethical approval of this study was obtained from the Institutional Review Board (IRB) of Myungsung Medical College. The participants of the study was informed about the purpose of the study and provided their written consent. At the end of the interview, the data collectors have provided information about the importance of HPV vaccination. All methods were carried out in accordance with relevant guidelines and regulations.

## Results

### Socio-demographic characteristics

Four hundred twenty-two (422) participants responded completely to the interview (98.1% response rate) and included in the analysis. The mean age of the study participants was 39.0 years (SD ± 9.9). Majority 362 (85.8%) of the study participants were females and 351(83.2%) married (Table 1).

**Table 1:** Socio-demographic characteristics of parents of daughters in Akaki Kality sub city, Addis Ababa, Ethiopia, 2021

Variables		Number (%)
Age in years	≤ 30	84 (19.9)
	31–59	313 (74.2)
	≥ 60	25 (5.9)
Sex	Male	60 (14.2)
	Female	362 (85.8)
Occupation	Employed	263 (62.4)
	Unemployed	36 (8.5)
	Housewife	123 (29.1)
Marital Status	Single	11 (2.6)
	Married	351 (83.2)
	Widowed	22 (5.2)
	Divorced	38 (9.0)
Education level	Not able to read and write	47 (11.1)
	Primary and secondary	255 (60.4)
	Diploma and above	120 (28.5)
Monthly income	≤ 3200 ETB (≤ 100 USD)	163 (38.6)
	> 3200 ETB (> 100 USD)	259 (61.4)
Number of children	1 girl	388 (91.9)
	2 girls	34 (8.1)
Religion	Christian	362 (85.8)
	Muslim	60 (14.2)
School type	Government school	238 (56.4)
	Private school	171 (40.5)
	Not going to school	13 (3.1)

### Knowledge of cervical cancer and HPV

The mean knowledge of cervical cancer score was 11.79 (SD ± 1.17). Overall 385 (91.2%) have heard about cervical cancer; out of which majority of them 318 (82.4%) got the information from mass media. However, only 25 (5.9%) knew that having multiple sexual partners is a risk, 16 (3.8%) and 11 (2.6%) knew that early initiation of sexual intercourse and HPV infection are risk factors of cervical cancer, respectively. Majority of the participants, 344 (81.5%), knew that the cancer is deadly and only 91 (21.6%) said women in the age group between 31 and 49 years are commonly affected. Overall, 246 (58.3%) had good knowledge on cervical cancer.

The mean score for knowledge of HPV was 4.66 (SD ± 0.45). Out of the study participants, only 88 (21%) have heard about HPV before, of which majority 36 (63.0%) of them got the information from the mass media (TV/Radio). Only 38 (9.0%) of the parents know that HPV can be sexually transmittable. Only 47 (11.0%) of the study participants believe that HPV infection causes cervical cancer. Over all, only 119 (28%) of the study participants had good knowledge about HPV.

### Knowledge, attitude and acceptance of HPV vaccine

The mean score for knowledge of HPV vaccine was 11.62 (SD ± 2.33). Nearly three-fourths (308, 73%) of the participants have heard about HPV vaccine with a large portion of them (260, 84.4%) believing that it prevent cervical cancer. The major portion of the participants (240, 77.9%) heard about HPV vaccine from mass media. About a quarter (94, 22.3%) of participants think vaccination is the only way of cervical cancer prevention. Only 42(10%) knew that the vaccine is given for both male and female.

The mean score for attitude towards the vaccine was 26.0 (SD ± 2.55). Out of the study participants, 75 (17.8%) were concerned about the side effects of the vaccination, out of which 35 (46.7%) of them mentioned that they fear infertility as a side effect of the vaccine. Over all, about two-thirds (268, 63.5%) of the study participants had positive attitude towards the vaccine.

### Acceptability of HPV vaccine

A substantial proportion of the study participants (n = 398, 94.3%; 95% CI 91.9–96.2%) were willing to vaccinate their daughters for HPV. Distribution of the HPV vaccine acceptability with the knowledge and attitude of the parents towards cervical cancer, HPV virus and HPV vaccine is presented in the Table 2.

**Table 2:** Distribution of the HPV vaccine acceptability with the knowledge and attitude of the parents in Akaki Kality sub city, Addis Ababa, Ethiopia, 2021

Variables	Accept vaccine	
	Yes (%)	No (%)
*Knowledge of HPV*		
Good Knowledge	107 (26.9)	12 (50.0)
Poor knowledge	291 (73.1)	12 (50.0)
*Knowledge of cervical cancer*		
Good Knowledge	232 (50.3)	14 (58.3)
Poor knowledge	166 (49.7)	10 (41.7)
*Attitude towards HPV vaccine*		
Positive Attitude	200 (50.3)	4 (16.7)
Negative Attitude	198 (49.7)	20 (83.3)
*Knowledge of vaccine*		
Good Knowledge	239 (60.1)	7 (29.2)
Poor knowledge	159 (39.9)	17 (70.8)

### Factors associated with acceptability of HPV vaccine

In the bivariate analysis, variables with *p* value < 0.25 include: marital status, income, knowledge of vaccine, attitude towards the vaccine, and knowledge of HPV.

In the further multi-variable analysis (Table 3), vaccine acceptability was associated with monthly income, knowledge of HPV, knowledge of vaccine, and attitude towards the vaccine. Accordingly, the odds of HPV vaccine acceptability among those parents of daughters with monthly income > 3200 ETB was 2.5 times (aOR = 2.48, 95% CI 1.08–6.34) higher as compared to those with parents of daughters with monthly income ≤ 3200 ETB. The odds of HPV vaccine acceptability among those parents of daughters with adequate knowledge on HPV (aOR = 2.32, 95% CI 1.56–4.87) and the vaccine (aOR = 2.24, 95% CI 1.12–8.60) was two times higher than their counterparts. Similarly, the odds of HPV vaccine acceptability among those parents of daughters with positive attitude towards the vaccine was five times (aOR = 5.03, 95% CI 1.63–9.56) higher as compared to those with negative attitude.

**Table 3:** Multi-variable analysis showing factors associated with vaccine acceptability among parents/guardians of daughters in Addis Ababa, Ethiopia, 2021

Variables	Accept vaccine		cOR (95% CI)	aOR (95% CI)	*P* value
	Yes (%)	No (%)			
*Marital status*					
Living with their partner	334 (83.9)	17 (70.8)	2.14 (0.86–5.39)	1.53 (0.71–4.63)	0.41
Not living with partner	64 (16.1)	7 (29.2)	1	1	
*Monthly income*					
> 3200 ETB (> 100 USD)	250 (62.8)	9 (37.5)	2.82 (1.20–6.59)	2.48 (1.08–6.34)	0.04
≤ 3200 ETB (≤ 100 USD)	148 (37.2)	15 (62.5)	1	1	
*Knowledge of HPV*					
Good knowledge	107 (26.9)	12 (50.0)	2.25 (1.52–4.56)	2.32 (1.56–4.87)	0.0001
Poor knowledge	291 (73.1)	12 (50.0)	1	1	
*Attitude*					
Positive attitude	200 (50.3)	4 (16.7)	5.84 (2.27–10.07)	5.03 (1.63–9.56)	0.001
Negative attitude	198 (49.7)	20 (83.3)	1	1	
*Knowledge of vaccine*					
Good knowledge	239 (60.1)	7 (29.2)	3.65 (1.48–9.04)	2.24 (1.12–8.60)	0.001
Poor knowledge	159 (39.9)	17 (70.8)	1	1	

## Discussion

The principal finding of this study showed that although the participants of the study had limited knowledge about HPV vaccine, majority of individuals were willing to vaccinate their child. This acceptance rate was consistent with the study findings of Nigeria (88.9%) [17], and Tanzania (93%) [21], but higher than rates noted in a South African (80%) study [12], Gondar (Northern Ethiopia) study (81.3%) [10], and Bench-Sheko zone, south-west Ethiopia (79.5%) study [15]. The highest vaccine acceptance rate in the present study could be partly due to the current national campaign for HPV vaccination in Ethiopia. During the data collection for this study, school based immunization was being given in the sub city in a campaign basis and the parents have heard and made their decision about the vaccine. The high acceptance of HPV vaccine in our study could be considered as an opportunity to scale-up the school-based HPV vaccines in all parts of the country. The variation in the acceptance rate from the study conducted in Gondar and Bench-Sheko zone could be partly due to the difference in socio-demographic characteristics of the participants. Our study participants were urban residents living in the capital with a greater access to health-based information and expected high level of health literacy as compared to other parts of the country.

However, more than four out of ten (41.7%) participants in our study had inadequate knowledge on cervical cancer, which is comparable with the study report of Nepal (46.7%) [3], but higher than the study report of UAE (21.7%) [14]. The discrepancy in the level of knowledge might reflect the health literacy disparities that could be associated with the socio-economic developments of the countries [24, 25]. The findings of lower knowledge, but higher acceptance in our study have public health implications. Since the study was conducted in the week when there was a campaign on HPV vaccination, people were exposed to mass media messages and willing to accept the vaccination. However, their willingness might not be sustainable and not reflect the usual trend, as it was not emanated from their in-depth knowledge and understanding of cervical cancer and its burden [26, 27]. Therefore, it is paramount important to design sustainable programs to educate the general public about cervical cancer prevention and control activities, including the HPV vaccination to ensure the sustainability of the interventions.

Of note, about a quarter of the study participants have never heard about HPV vaccination and more than one-third had negative attitude towards it. Generally, acceptance of the health intervention is directly related to the knowledge and attitude of the recipients of the intervention [17, 19]. In a similar manner, in our study, knowledge and attitude towards the HPV vaccine were found to be determinant factors of the parents’ acceptance of the HPV vaccination for their daughters. These findings of the study are in line with findings of other studies conducted in Ethiopia [10] and France [22]. Consistent to other studies, the commonest source of information for the HPV vaccination in the present study was mass media. Mass media messages are considered as reliable source of information by the community in Ethiopia [28, 29]. Therefore, the findings of this study underscore the need for concerted efforts to design appropriate mass media messages pertaining to cervical cancer and HPV vaccination to address the gap in knowledge and attitudes towards HPV vaccination in the general population.

Consistent to other study findings, monthly income was found to be associated with the acceptance of HPV vaccine. Those parents with lower monthly income were not willing to vaccinate their daughter as compared to those with higher income [30, 31]. This might be due to their low level of exposure to the mass media messages, low level of health literacy, and low level of exposure to the effects of globalization and urbanization. This finding of the study calls for interventions that give special considerations for addressing the gaps of knowledge on cervical cancer and HPV vaccination among the low socio-economic population.

This study was conducted in a community by using a representative sample and using a standardized tool. Moreover, it is the first study from the capital city of Ethiopia to comprehensively describe knowledge and acceptability of HPV vaccination among the parents. However, it might be limited due to the vaccination campaign which was started days before we begun the data collection. Moreover, it might be also affected by recall and social desirability bias.

## Conclusions

Although the overall HPV vaccine acceptance was high, the level of knowledge and positive attitude towards cervical cancer and HPV vaccination were found to be limited. Higher monthly income, good knowledge on HPV and the vaccine, and positive attitude towards the vaccine were associated with acceptance of HPV vaccination. To ensure a sustainable acceptance of HPV vaccination, it is crucial to increase the community awareness in a sustainable manner.

## Data Availability

Data are available upon reasonable request from the corresponding author.

## Abbreviations

aOR: Adjusted odds ratio
CI: Confidence interval
cOR: Crude odds ratio
ETB: Ethiopian birr
HPV: Human papillomavirus
OR: Odds ratio
SD: Standard deviation
UAE: United Arab Emirates
